# Non-invasive detection of *EGFR* mutations by cell-free loop-mediated isothermal amplification (CF-LAMP)

**DOI:** 10.1038/s41598-020-74689-3

**Published:** 2020-10-16

**Authors:** Srividya Arjuna, Rajesh Venkataram, Pandyanda Nanjappa Dechamma, Gunimala Chakraborty, Nishith Babu, Audrey D’Cruz, Giridhar Belur Hosmane, Anirban Chakraborty

**Affiliations:** 1 Division of Molecular Genetics and Cancer, Nitte University Centre for Science Education and Research (NUCSER), Nitte (Deemed to be University), Kotekar-Beeri Road, Deralakatte, Mangaluru, 575018 India; 2 Department of Pulmonary Medicine, K S Hegde Medical Academy, Nitte (Deemed to be University), Mangaluru, 575018 India; 3 Department of Health Dentistry, A B Shetty Memorial Institute of Dental Sciences, Nitte (Deemed to be University), Mangaluru, India

**Keywords:** Cancer, Genetics, Molecular biology

## Abstract

Targeting epidermal growth factor receptor (EGFR) through tyrosine kinase inhibitors (TKI) is a successful therapeutic strategy in non-small cell lung cancer. However, the response to TKI therapy depends on specific activating and acquired mutations in the tyrosine kinase domain of the *EGFR* gene. Therefore, confirming the *EGFR* status of patients is crucial, not only for determining the eligibility, but also for monitoring the emergence of mutations in patients under TKI therapy. In this study, our aim was to develop a cost effective, yet sensitive, technique that allows the detection of therapeutically-relevant *EGFR* hotspot mutations at isothermal conditions in a non-invasive manner. Previously, we developed an allele-specific loop-mediated isothermal amplification (AS-LAMP) assay for screening germline and somatic de novo T790M EGFR mutation in lung cancer patients. In this study, we used cell free DNA as a template in AS-LAMP assay (CF-LAMP) for non-invasive detection of two hotspot EGFR mutations (T790M, and L858R) and compared its efficiency with ultrasensitive droplet digital PCR (ddPCR) assay. The results of CF-LAMP assay were consistent with those obtained in ddPCR assay, indicating the robustness of the method. CF-LAMP may serve as a valuable and cost-effective alternative for liquid biopsy techniques used in molecular diagnosis of non-small cell lung cancer.

## Introduction

Lung cancer represents one of the major causes of cancer-related deaths worldwide and non-small cell lung cancer (NSCLC) is the predominant subtype, accounting for approximately 85% of the cases^1^. Targeting tyrosine kinases through inhibitors (TKI therapy), is an effective treatment option in NSCLC. However, specific mutations in the epidermal growth factor receptor (EGFR) gene influence the response of patients to TKI therapy^2^. Within EGFR, exons 18–24 code for the tyrosine kinase domain. However, mutations that interfere with the response to TKIs are restricted to exons 18–21^3,4^. Among the frequently observed activating mutations that confer sensitivity to TKIs, small in-frame deletions in exon 19 and point mutations in exon 21 causing a leucine to arginine substitution at codon 858 (L858R) are the “hotspots”, comprising about 85–90% of all such activating mutations^3^. The remaining 10% are uncommon activating mutations, of which G719X, point mutation at codon 719 that results in substitution of glycine with alanine (G719A), cysteine (G719C) and serine (G719S), accounts for approximately 5% of the mutations^4,5^. In-frame duplications and/or insertions in exon 20 account for the remaining 5% of EGFR TK activating mutations^4^. On the other hand, mutations associated with TKI resistance are concentrated in exon 20, with point mutation at codon 790 resulting in substitution of tyrosine with methionine (T790M) accounting for nearly 50% of all such mutations^3^. Therefore, a routine molecular testing of NSCLC generally includes four hotspot mutations, three associated with drug sensitivity (G719X, Exon 19 deletion, and L858R) and one associated with drug resistance (T790M).

Liquid biopsy is one of the most rapidly advancing techniques in cancer management, which allows the detection of oncogene mutations through non-invasive techniques using blood or any other body fluid^6^. Cancer cells release tumor-derived DNA, called circulating tumor DNA (ctDNA), into the bloodstream, which can be extracted from plasma or serum^7^. Although patients with cancer usually have higher average plasma/serum levels of ctDNA, they are often present in fragmented form at very low concentrations^8^. Digital PCR (dPCR) is the third generation and the most advanced PCR technique that permits highly sensitive genotyping and absolute quantification of mutant copies in ctDNA^9,10^. While dPCR is highly accurate, it is cost-intensive, time consuming, and requires sophisticated instruments and trained personnel. Moreover, in economically-marginalized countries, the high cost of a dPCR test makes it unaffordable for many patients. Therefore, development of novel and sensitive, yet cost-effective, techniques that enable the detection of mutations in a non-invasive manner is always desirable. One such diagnostic technique is Loop Mediated isothermal Amplification technique (LAMP). LAMP assay relies on isothermal amplification of the desired gene fragment, using four primers, two outer (F3 and B3) and two inner (FIP and BIP) that mark six regions within the target sequence. The assay works on auto cycling strand displacement activity of target DNA by *Bst* polymerase, and results in the formation and accumulation of stem loop DNA^11^. In this study, we have developed novel, cell free DNA-based allele specific loop-mediated isothermal amplification (CF-LAMP) assay for screening two clinically relevant mutations, T790M (C>T) point mutation in exon 20 and L858R (T>G) point mutation in exon 21 of *EGFR* gene. Here we propose CF-LAMP as a cost-effective alternative to liquid biopsy techniques used in molecular testing of non-small cell lung cancer, particularly in resource-constraint settings.

## Results

### Sample preparation for CF-LAMP assay

**Figure 1:**
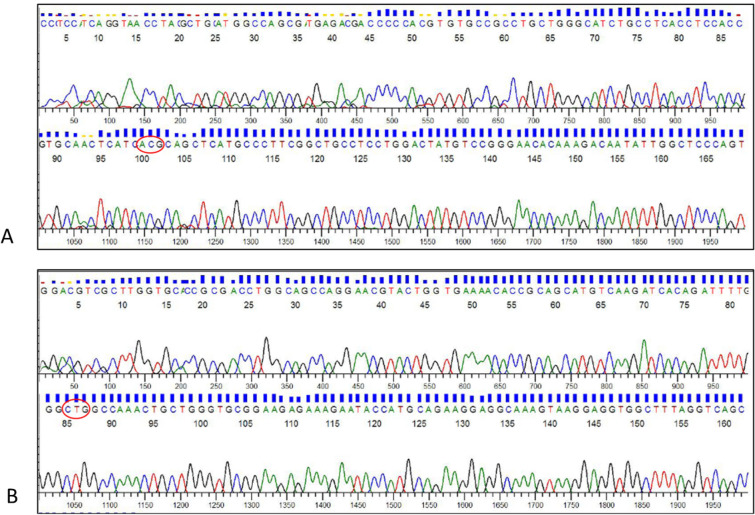
Partial sequence of PCR amplified exon 20 (**A**), exon 21 (**B**) of *EGFR* gene using cfDNA as template. The sequence highlighted in red circle indicates the codon for amino acid Tyrosine at 790th position in exon 20 (**A**) and codon for amino acid Lysine at 858th position in exon 21 (**B**) of *EGFR* gene.

**Figure 2:**
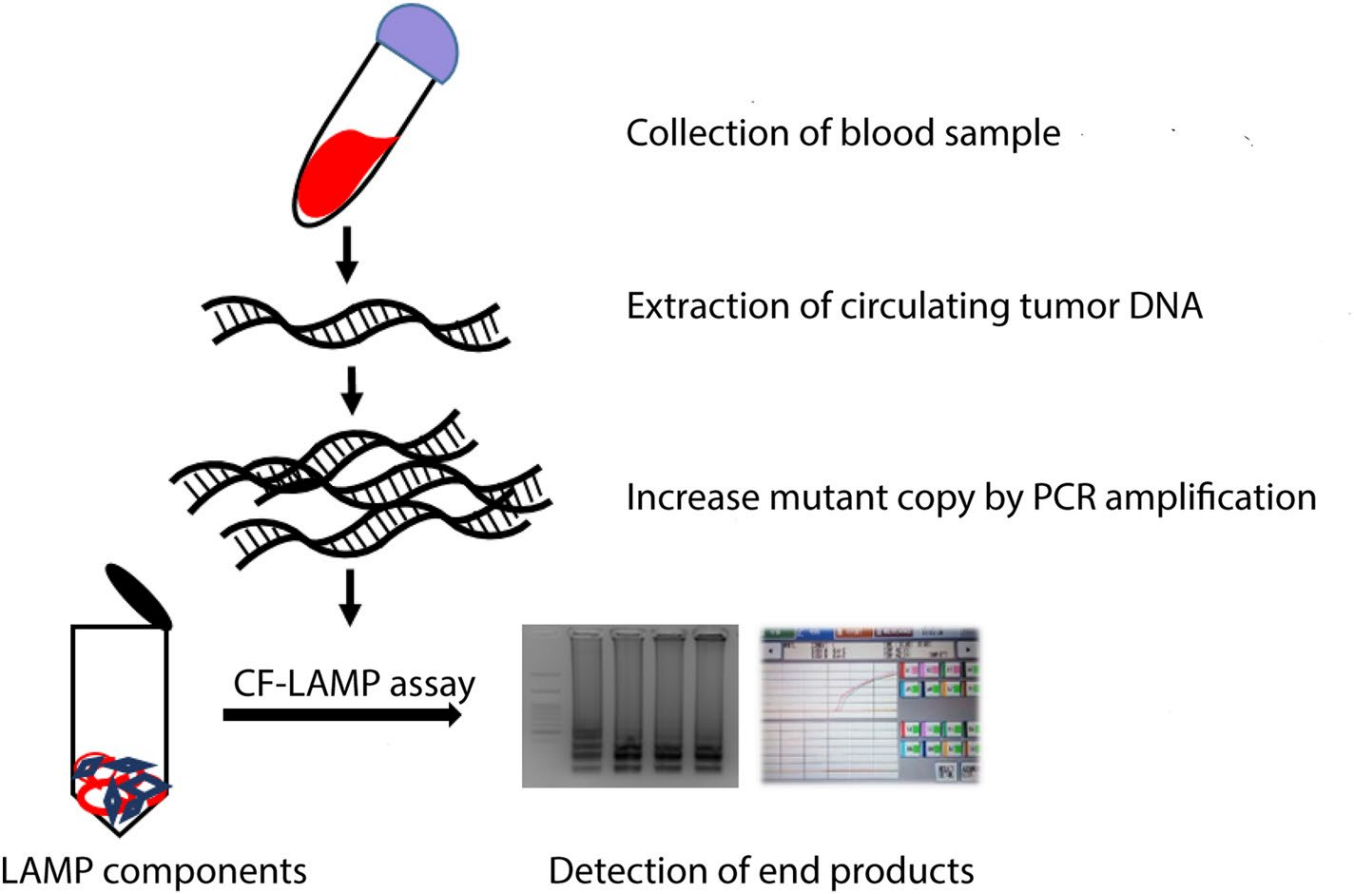
Schematics of the work flow of CF-LAMP assay.

As cfDNAs are present as small fragments with low copy number in the plasma, we enriched the copies of target cfDNA by performing an initial PCR amplification using the outer primers designed for LAMP (F3, B3). To confirm whether the outer LAMP primers could accurately amplify the gene and the region of interest, we sequenced a few randomly selected amplified cfDNA products. Direct sequencing revealed that the amplified cfDNA products were indeed fragments of exon 20 and 21 of *EGFR* gene (Fig. 1). The amplified cfDNA was then used as template for CF-LAMP assay. A schematic of the CF-LAMP work flow is shown in Fig. 2.

### CF-LAMP de novo T790M and L858R mutation analysis

We screened a total of 45 samples for de novo T790M mutation. Among the 45 samples, 41 (~ 91%) were of non-small cell lung cancer type and 4 (~ 9%) were small cell lung cancer. CF-LAMP identified 19 samples as positive for the target mutation while the remaining 26 were negative. In case of L858R, we screened 28 samples. Of these 28, 25 (~ 90%) were of non-small cell lung cancer type and 3 (~ 10%) belonged to small cell lung cancer. CF-LAMP of L858R indicated 25 as positive for L858R mutation. The remaining 3 samples showed non-specific amplification and subsequent repetition showed inconsistent results. The clinical details of the samples used are mentioned in Supplemental Tables 1 and 2.

### Validation of CF-LAMP results by droplet digital PCR (ddPCR)

**Figure 3:**
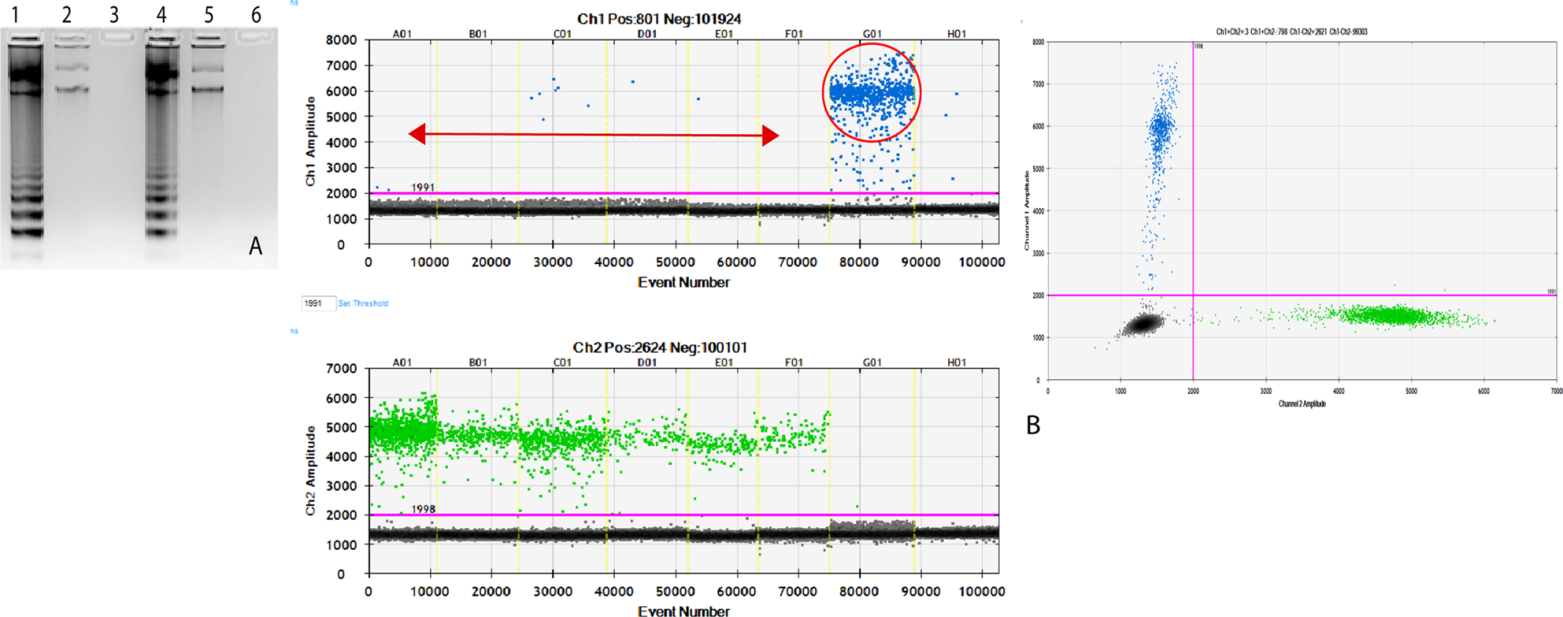
Screening of de novo T790M mutation by CF-LAMP and ddPCR (**A**, **B**). Representative images of both the assays are shown here. (**A**) T790M CF-LAMP assay. Lane1: Positive control (Plasmid DNA harbouring T790M mutation), Lane2-5: Patient’s cell-free DNA samples. Lane 6: Negative control. The cropped gel image is shown here. The full-length gel is included as Supplemental Image 1. **(B)** T790M ddPCR assay. Snapshot of 1D and 2D plots obtained from the QuantaLife software in QX200 ddPCR platform. The assay included a set of primers and two competitive probes, one labelled with FAM (T790M mutant allele, Channel 1) and another with HEX (T790M wild type allele, Channel 2). Lanes A01 to F01 indicate patient-derived cfDNA (red double headed arrow). The blue droplets (FAM positive) indicate the presence of mutant copies in the sample (column C01). Green droplets (HEX positive) indicate the wild-type copies present in all lanes from A01 to G01. The black droplets below the threshold line (magenta) are the negative droplets having no DNA. The column G04 is the positive control (T790M mutant plasmid DNA) and the column H04 is no-template control.

**Figure 4:**
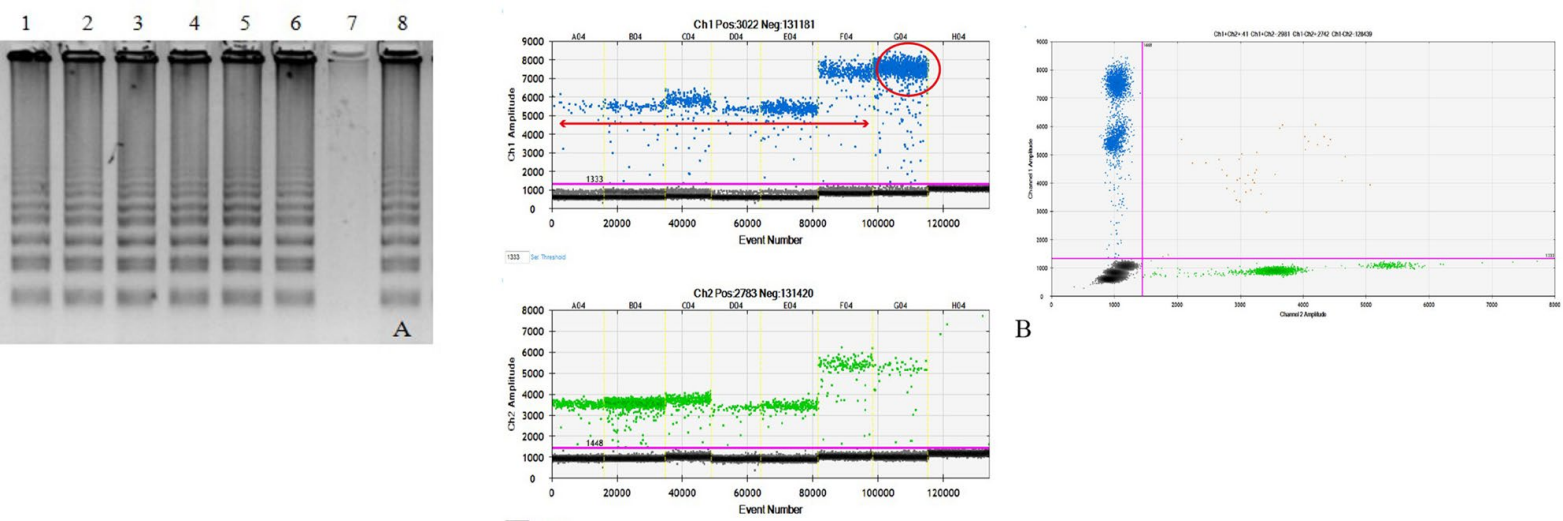
Screening of L858R mutation by CF-LAMP and ddPCR (**A**, **B**). Representative images of both the assays are shown here. (**A**) L858R CF-LAMP assay. Lane1: Positive control (Plasmid DNA harbouring L858R mutation), Lane2-8: Patient’s cell-free DNA samples. The cropped gel image is shown here. The full-length gel is included as Supplemental Image 2 (**B**) L858R ddPCR assay. Snapshot of 1D and 2D plots obtained from the QuantaLife software in QX200 ddPCR platform. The assay included a set of primers and two competitive probes, one labelled with FAM (L858R mutant allele, Channel 1) and another with HEX (L858R wild type allele, Channel 2). Lanes A04 to F04 indicate patient-derived cfDNA (red double headed arrow). The blue droplets (FAM positive) indicate the presence of mutant copies in the sample (column C01). Green droplets (HEX positive) indicate the wild-type copies present in all lanes from A01 to G01. The black droplets below the threshold line (magenta) are the negative droplets having no DNA. The column G04 is the positive control (L858R mutant plasmid DNA) and the column H04 is no-template control.

To validate the results of CF-LAMP assay, we performed ddPCR on 20 samples each for T790M and L858R mutation, randomly selected from those already screened by CF-LAMP. Droplet digital PCR is the third generation in the PCR technology and is highly precise with extremely low false-positive rate. The basic principle of rare mutation detection by ddPCR is the use of a single set of primers but two competitive probes, one for the wild type allele (labelled with HEX) and another for the mutant allele (labelled with FAM). The method provides an absolute measure of target DNA molecules and works on partitioning effect of several thousand water–oil emulsion droplets where each droplet is subjected to amplification, resulting in data with great precision and sensitivity. In case of de novo T790M mutation, 19 of 20 (95%) samples showed concordance between CF-LAMP and ddPCR. For L858R, all the 20 samples (100%) showed similar results in both the techniques (Table 1). Z-test revealed no significant difference between CF-LAMP and ddPCR for de novo T790M mutation with p value of 0.7515. Representative images of CF-LAMP and ddPCR are shown in Figs. 3 and 4.

**Table 1:** Comparison of results obtained from CF-LAMP and ddPCR assays.

EGFR mutation	CF-LAMP		ddPCR	
	Positive	Negative	Positive	Negative
Exon 20 T790M (N = 20)	9	11	10	10
Exon 21 L858R (N = 20)	20	0	20	0

## Discussion

Direct sequencing is considered the gold standard for identification of gene mutations. However, alternative methods that can detect the presence of target mutations without sequencing are now considered as valid methods in molecular diagnosis. Initially developed as a technique for rapid identification of infectious agents, LAMP has undergone extensive modifications in recent years^12^. AS-LAMP, which is an important variant of traditional LAMP, is now being appreciated as an effective alternative in SNP identification^11^. Previously, we developed AS-LAMP technique for screening germline and somatic de novo T790M mutation using blood and tissue DNA^13^. In this study, the focus was to develop a non-invasive technique for detecting clinically-relevant EGFR mutations using cfDNA. Validation of CF-LAMP results by ultrasensitive ddPCR indicated a high level of consistency between the two techniques.

Liquid biopsy relies on circulating tumor-derived nucleic acids (ctNA; DNA or RNA), which are a subset of cell free nucleic acids^14^. The cfDNAs are fragmented DNAs that are released into the bloodstream as a result of various phenomenon like necrosis and apoptosis. Patients with cancer usually have higher average plasma/serum levels of cfDNA, which make them good surrogate for mutation screening traditionally done by tissue biopsy^15^. The prognostic and the predictive applications of liquid biopsy are most relevant in lung cancer management. However, despite recent advancements in the analysis of cfDNA, many challenges still exist in liquid biopsy. One of the limitations is the insufficiency of cfDNA in the amount of blood^15^. Another critical factor is the cost of the test, which makes it unaffordable for majority of the population in lower income groups. A typical liquid biopsy is cost-intensive and requires the digital PCR platforms. In addition, the availability of technical personnel with sound scientific knowledge on the use of the equipment is another important pre-requisite. In contrast, the CF-LAMP developed in this study, is a simple method, which can be easily implemented in a resource-constrained set up. The results obtained through CF-LAMP showed almost 100% concordance with those obtained by the ultrasensitive ddPCR, clearly indicating the robustness of the developed assay. The fact that the method works at isothermal conditions, makes it feasible for use as a potential IVD technique in routine hospital set up.

We screened cfDNA from lung cancer patients for two clinically-relevant mutations namely T790M and L858R in tyrosine kinase domain of EGFR gene. Given the fact that the method works at isothermal conditions and relies on amplification of the desired gene fragment using four specific primers, the method can be used for screening other clinically relevant, but uncommon, mutations associated with TKI resistance or sensitivity. For example, D761Y, a T790M-like secondary mutation in exon 19 of EGFR associated with TKI resistance, or V765A and T783A substitutions in exon 20, and L861Q substitutions in exon 21 associated with drug sensitivity^3^. Our CF-LAMP assay showed the presence of de novo mutation in 42% of the cases tested. Although T790M mutation is generally considered as an acquired mutation that emerges during the TKI treatment, primary de novo EGFR T790M somatic mutations have been reported in patients that carry dual or multiple EGFR mutations^16^. Moreover, it has also been reported that most T790M are often present in the same EGFR allele and on treatment, these mutations undergo selection and enrichment giving rise to drug resistance^17^.

L858R is one of the most common activating mutations that confers sensitivity to TKI and accounts for about 41% of observed EGFR mutations in NSCLC^18^. In our study, we observed a staggering 89% of the cases to be positive for this mutation. This was in expected lines as L858R is usually present in higher percentage in Asian population, particularly in women never smokers with adenocarcinoma^19^. We couldn’t get conclusive results on three samples by CF-LAMP. Interestingly, these three samples were negative by ddPCR. Non-specific amplifications in L858R mutation screening has been reported in a previous study^20^.

The liquid biopsy techniques currently in use are highly cost-intensive. By targeting two hotspot mutations (T790M and L858R) that interfere with TKI therapy in non-small cell lung cancer, we have demonstrated that CF-LAMP is an effective and a cost-effective alternative for liquid biopsy. The developed assay could be used in a resource-constraint setting for molecular testing of non-small cell lung cancer. The method described here is rapid and easy to perform. The level of sensitivity is almost equal to ddPCR, which is the most advanced and sensitive molecular method available at present. However, unlike ddpCR, absolute quantification of the mutant copies in a sample is not possible in a conventional CF-LAMP method. The method developed here is a semi-quantitative assay aimed at detecting the presence or absence of mutated cfDNA in non-small cell lung cancer patients. Future efforts could be directed towards improvising this assay for quantification of the mutant DNA copies using probe-based techniques. In a previous LAMP-based study, the detection limit was measured by the fluorescence intensity of a ds-DNA dye at real time through a fluorometer using tenfold serial-dilution of constructed plasmids that harbored specific polymorphisms^21^.

To conclude, traditional biopsies are invasive, expensive and time consuming. Moreover, it is impossible to perform in critically ill patients. Repeated biopsies during the treatment regimen are hard on patients, both physically and emotionally. The CF-LAMP developed here could be effectively utilized as a non-invasive molecular tool, not only for diagnostic, but also for prognostic and predictive applications of liquid biopsy. For instance, using CF-LAMP, a clinician can easily determine the suitability of the patient for TKI therapy by checking the status of sensitizing *EGFR* mutations in tumor-derived DNA from peripheral blood. Similarly, CF-LAMP would allow the clinicians to use the liquid biopsy approach for monitoring the emergence of resistance in patients undergoing TKI therapy. Considering the fact that the CF-LAMP assay developed here includes a pre-amplification step, a high cfDNA input is required for this technique. Here we used a concentration that is generally recommended for amplification of genomic DNA and used the pre-amplified template in CF-LAMP assays at a concentration, which is equivalent to that used in singleplex ddPCR assays. Previously, using genomic DNA, we have estimated that the amount of input material for AS-LAMP could be as low as 10 fg^13^. Although NGS-based liquid biopsy assays require less DNA input, they are cost-intensive and technically demanding, which make them unsuitable in clinical settings. On the other hand, ddPCR can be adopted in a clinical setting but they require significant amount of input DNA. Thus, in order to determine the feasibility of the use of CF-LAMP in clinical practice, it would be necessary to estimate the limit of detection of input material (cfDNA) during the pre-amplification step, which in turn would allow the detection of many mutations using a small amount of cfDNA. In this study, we have evaluated the applications of CF-LAMP in screening point mutations in circulating cfDNA. However, considering that the most important factor is the design of primers for a target mutation, this technique would allow the screening for other kinds of activating or acquired mutations, including deletions or insertions that are observed in patients. The CF-LAMP assay has tremendous potential as a cost-effective alternative to the cost-intensive liquid biopsy techniques currently used in IVD.

## Methods

### Cell-free DNA extraction

Seventy-three patients with confirmed diagnosis of lung carcinoma based on histopathological and radiological features were included in the study. Five ml of peripheral blood was collected in EDTA-coated vacutainers after obtaining individual informed consents. Plasma was isolated within 2 h of collection by centrifuging the blood samples at 3500 rpm for 20 min at 4 ℃. The extraction of cell-free DNA (cfDNA) from plasma was performed by using QIAamp circulating nucleic acid kit (Qiagen, Germany) as per the manufacturer’s instructions. The quantity of the cfDNA extracted was estimated by measuring the concentration at 260 nm wavelength using a nanophotometer (Implen, Germany). The extracted cfDNAs were stored at − 20 °C till further use. The study was approved by the Central Ethics Committee of Nitte (Deemed to be University) and informed consents were obtained from all the patients.

### CF-LAMP primer design

A typical LAMP assay utilizes four primes, namely the forward and backward inner primers (FIP & BIP) and the forward and backward outer primers (F3 and B3). These primers bind to six distinct regions in the target gene. LAMP primers were designed to amplify two different fragments of exon 20 and exon 21 of *EGFR* gene. The CF-LAMP assays developed in this study relied on the principle of allele-specific LAMP (AS-LAMP) technique, which can differentiate between mutant and wild-type DNA based on changes in primer sequences. In AS-LAMP assays, only the BIP primer changes for mutant and wild type sequence while the rest of the primers remain same. Thus, for CF-LAMP assays, the modifications were made in the 5′ end of BIP for each target mutation (T790M and L858R) in exon 20 and 21 respectively. The BIP primer designed were mutant-specific which allowed amplification only in presence of mutant DNA and not wild-type DNA. The primers were designed using the primer explorer V4 software (https://primerexplorer.jp/e/), Eiken Chemical, Tokyo, Japan. The sequences of the primers are available on request.

### Optimization of LAMP assay parameters

The optimization of time, temperature, specificity and sensitivity was done using commercial plasmid DNA constructs (pBabe EGFR T790M, #32070; EGFR WT, #11011) procured from Addgene (https://www.addgene.org/) following the protocol described earlier^13^. For L858R optimization, plasmid DNA harboring the target mutation were generated using site-directed mutagenesis assay (New England Biolabs, USA) with EGFR WT plasmid (#11011, Addgene) as the template. In our previous study^13^, we have shown that the limit of detection in AS-LAMP assay is 10 fg of DNA.

### CF-LAMP assay

#### CF-LAMP protocol

CF-LAMP assay was carried out as per the manufacturer’s instructions (EIKEN chemicals, Japan). The master mix was prepared with the final reaction volume of 12.5 µl containing 10 µM of FIP, 3 µM of BIP and 0.5 µM of F3, B3 primers. For enrichment of the copies of target cfDNA, an initial PCR amplification was performed using 100 ng cfDNA as template in a 50 µl reaction cocktail. The PCR products were purified using column purification method (Qiagen, Germany) and the concentration was measured using a nanophotometer (Implen, Germany). For each sample, 30–50 ng of enriched cfDNA was used as template for LAMP experiments. The final detection was done by gel electrophoresis. All the experimental procedures were performed on ice.

#### Validation of CF-LAMP assays by droplet digital PCR

The CF-LAMP results were validated by the ultrasensitive droplet digital PCR (ddPCR) using the QX200 droplet digital PCR system (Bio-Rad, USA). In ddPCR, template cfDNA was used at a concentration of 60 ng/sample. PrimePCR ddPCR mutation assays validated for T790M and L858R were used and the methodology followed was as per the manufacturer’s instructions (Bio-Rad, USA).

### Statistical analysis

For comparison of the results obtained from CF-LAMP and ddPCR assays, a two-proportion Z test was performed and a P value of < 0.05 was considered statistically significant.

### Ethical approval and consent to participate

All procedures in this study that involved human participants were performed in accordance with the ethical standards of the institutional and/or national research committee and with the 1964 Declaration of Helsinki and its later amendments or comparable ethical standards.

### Informed consent

Informed consent was obtained from all individual participants included in the study.

## Supplementary information

Supplementary Information.
