# Supplementary material for: Non-invasive detection of EGFR mutations by cell-free loop-mediated isothermal amplification (CF-LAMP)
Source: Sci Rep. 2020 Oct 16;10:17559. doi: 10.1038/s41598-020-74689-3 (PMC7568567; doi:10.1038/s41598-020-74689-3)
Supplement: Supplementary file 1 — Supplementary Information. [file 41598_2020_74689_MOESM1_ESM.pdf]

Non-invasive detection of *EGFR* mutations by cell-free loop-mediated isothermal amplification (CF-LAMP)

1. Srividya Arjuna[#]
Division of Molecular Genetics and Cancer, Nitte University Centre for Science Education and Research (NUCSER), Nitte (Deemed to be University), Kotekar-Beeri Road, Deralakatte, Mangaluru-575018
Email: srimachar24@gmail.com

2. Rajesh Venkataram[#]
Department of Pulmonary Medicine, KS Hegde Medical Academy, Nitte (Deemed to be University), Mangaluru-575018
Email: rajdocmmc@gmail.com

3. Dechamma Pandyanda Nanjappa
Division of Molecular Genetics and Cancer, Nitte University Centre for Science Education and Research (NUCSER), Nitte (Deemed to be University), Kotekar-Beeri Road,Deralakatte, Mangaluru-575018
Email: divyadech@gmail.com

4. Gunimala Chakraborty
Associate Professor
Division of Molecular Genetics and Cancer, Nitte University Centre for Science Education and Research (NUCSER), Nitte (Deemed to be University), Kotekar-Beeri Road,Deralakatte, Mangaluru-575018
Email: gunimala@nitte.edu.in

5. Nishith Babu
Division of Molecular Genetics and Cancer, Nitte University Centre for Science Education and Research (NUCSER), Nitte (Deemed to be University), Kotekar-Beeri Road, Deralakatte, Mangaluru-575018
Email:nishibabu@gmail.com

6. Audrey D'Cruz
Professor and Head
Department of Public Health and Dentistry
A.B Shetty Memorial Institute of Dental Science, Nitte (Deemed to be University), Mangaluru-575018
Email:audreydcruz@nitte.edu.in

7. Giridhar Belur Hosmane
Professor and Head
Department of Pulmonary Medicine, KS Hegde Medical Academy, Nitte (Deemed to be University), Mangaluru-575018
Email: giridhar.belur@gmail.com

8. Anirban Chakraborty*
Professor
Division of Molecular Genetics and Cancer, Nitte University Centre for Science Education and Research (NUCSER), Nitte (Deemed to be University), Kotekar-Beeri Road, Deralakatte, Mangaluru-575018
Email: anirban@nitte.edu.in

# Contributed equally

*Corresponding author-
Anirban Chakraborty
Professor
Division of Molecular Genetics and Cancer, Nitte University Centre for Science Education and Research (NUCSER), Nitte (Deemed to be University), Kotekar-Beeri Road, Deralakatte, Mangaluru-575018
Email: anirban@nitte.edu.in
Phone: 0091-824-2204292

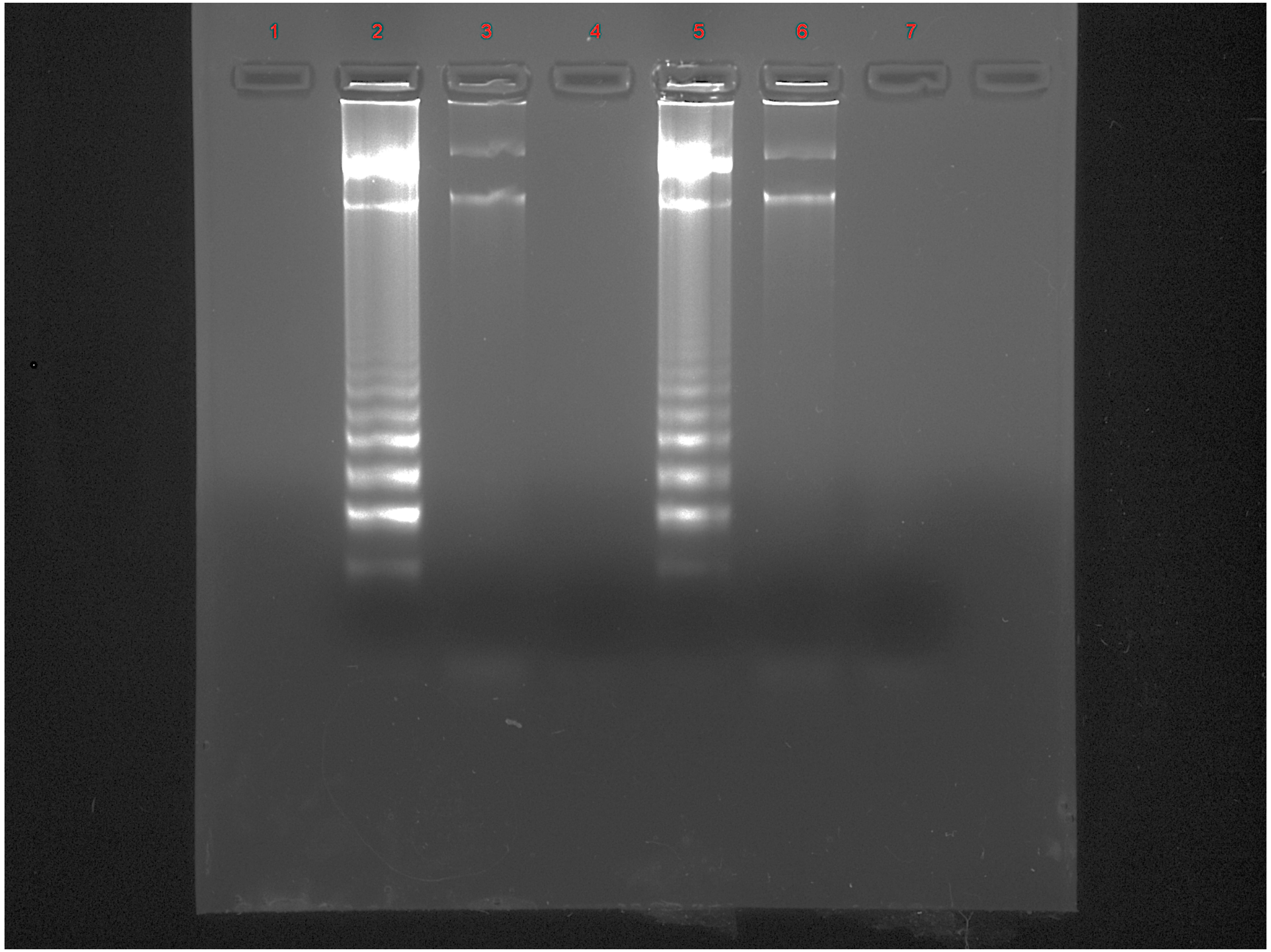

Supplemental Image 1: Full-length gel image of CF-LAMP assay carried out for T790M mutation.
Lane 1: Positive control. Lanes 2-6: patient cfDNA. Lane 7: Negative control

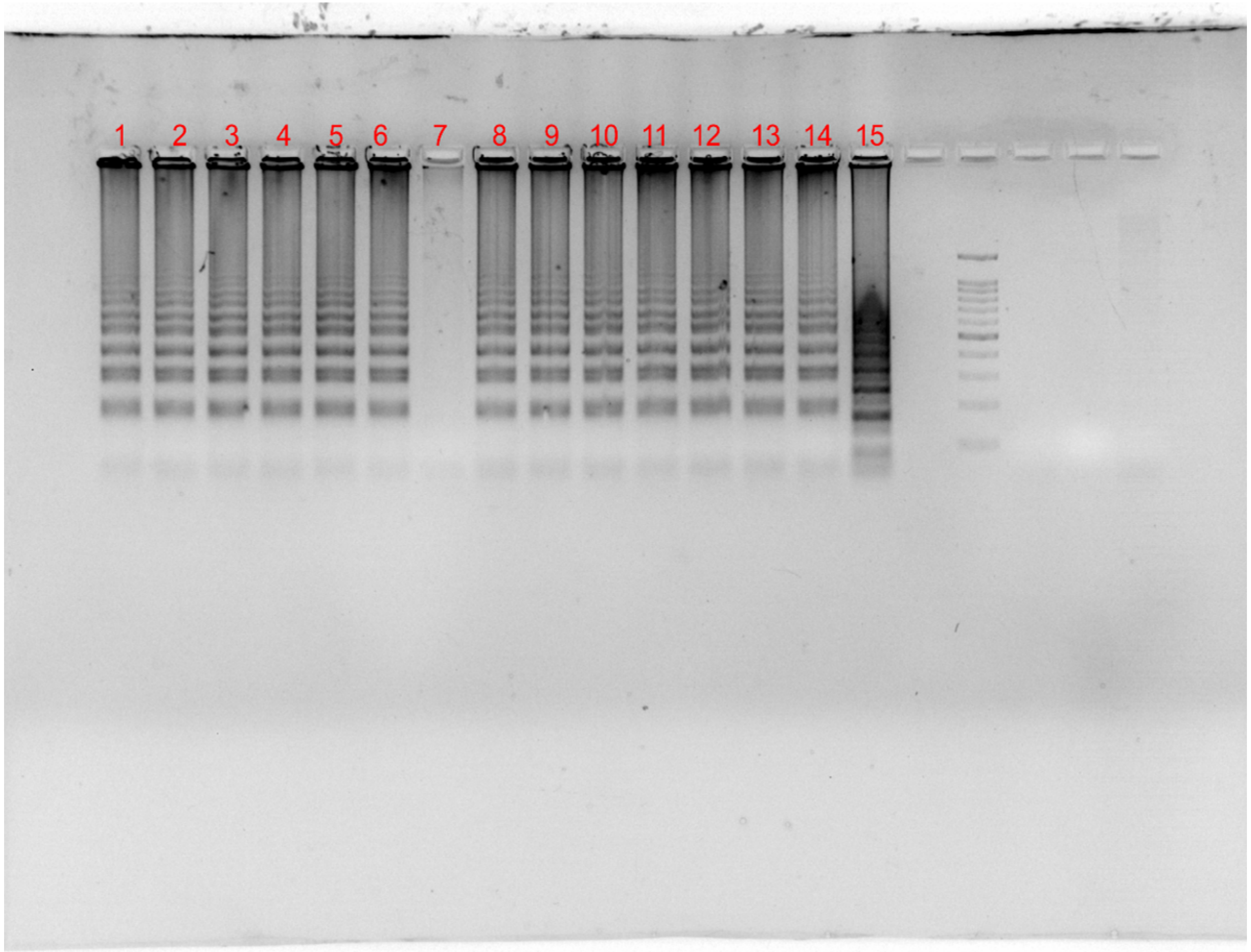

Supplemental Image 2: Full-length gel image of CF-LAMP assay carried out for L858R mutation.
Lane 1: Positive control. Lanes 2-15: patient cfDNA.

Supplemental **Table 1**: Clinical details of samples used for T790M mutation screening

SAMPLE ID	SMOKING STATUS	LUNG CANCER TYPE
LC 132	SMOKER	**NSCLC**
LC 113	SMOKER	**NSCLC**
LC 148	SMOKER	**NSCLC**
LC 165	SMOKER	**NSCLC**
LC 229	NON-SMOKER	**NSCLC**
LC 196	SMOKER	**NSCLC**
LC 169	SMOKER	**NSCLC**
LC 150	SMOKER	**NSCLC**
LC 141	SMOKER	**NSCLC**
LC 143	NON-SMOKER	**SCLC**
LC 157	NON-SMOKER	**NSCLC**
LC 175	SMOKER	**NSCLC**
LC 168	SMOKER	**NSCLC**
LC 214	NON-SMOKER	**NSCLC**
LC 174	NON-SMOKER	**NSCLC**
LC 208	NON-SMOKER	**NSCLC**
LC 239	SMOKER	**NSCLC**
LC 228	SMOKER	**NSCLC**
LC 226	NON-SMOKER	**NSCLC**
LC 222	NON-SMOKER	**NSCLC**
LC 194	SMOKER	**NSCLC**
LC 197	SMOKER	**NSCLC**
LC 200	SMOKER	**NSCLC**
LC 193	SMOKER	**NSCLC**
LC 243	NON-SMOKER	**NSCLC**
LC 135	SMOKER	**NSCLC**
LC 211	SMOKER	**NSCLC**
LC 131	NON-SMOKER	**NSCLC**
LC 183	SMOKER	**NSCLC**
LC 202	SMOKER	**NSCLC**
LC 146	SMOKER	**NSCLC**
LC 215	NON-SMOKER	**NSCLC**
LC 112	SMOKER	**SCLC**
LC 238	NOT AVAILABLE	**NSCLC**
LC 256	SMOKER	**NSCLC**
LC 170	NOT AVAILABLE	**NSCLC**
LC 142	NON-SMOKER	**SCLC**
LC 178	NON-SMOKER	**NSCLC**
LC 210	NON-SMOKER	**NSCLC**
LC 166	SMOKER	**NSCLC**
LC 198	SMOKER	**SCLC**
LC 152	SMOKER	**NSCLC**
LC 149	SMOKER	**NSCLC**
LC 250	SMOKER	**NSCLC**
LC 116	NON-SMOKER	**NSCLC**

NSCLC: Non-small cell lung cancer; SCLC: Small cell lung cancer

Supplemental **Table 2**: Clinical details of samples used for L858R mutation screening

SAMPLE ID	SMOKING STATUS	LUNG CANCER TYPE
LC 156	SMOKER	**NSCLC**
LC 201	SMOKER	**NSCLC**
LC 149	SMOKER	**NSCLC**
LC 112	SMOKER	**SCLC**
LC 116	NON-SMOKER	**NSCLC**
LC 137	SMOKER	**NSCLC**
LC 169	SMOKER	**NSCLC**
LC 211	SMOKER	**NSCLC**
LC 141	SMOKER	**NSCLC**
LC 196	SMOKER	**NSCLC**
LC 152	SMOKER	**NSCLC**
LC 174	NON-SMOKER	**NSCLC**
LC 125	SMOKER	**NSCLC**
LC 217	NON-SMOKER	**NSCLC**
LC 120	SMOKER	**NSCLC**
LC 111	SMOKER	**NSCLC**
LC 115	NON-SMOKER	**NSCLC**
LC 181	NON-SMOKER	**NSCLC**
LC 198	SMOKER	**SCLC**
LC 173	NON-SMOKER	**NSCLC**
LC 179	NON-SMOKER	**NSCLC**
LC 206	NON-SMOKER	**SCLC**
LC 238	SMOKER	**NSCLC**
LC 255	SMOKER	**NSCLC**
LC 208	NON-SMOKER	**NSCLC**
LC 228	SMOKER	**NSCLC**
LC 259	NON-SMOKER	**NSCLC**
LC 260	NON-SMOKER	**NSCLC**

NSCLC: Non-small cell lung cancer; SCLC: Small cell lung cancer
